# Chronic stress model simulated by salbutamol promotes tumorigenesis of gastric cancer cells through β2-AR/ERK/EMT pathway

**DOI:** 10.7150/jca.65403

**Published:** 2022-01-01

**Authors:** YanJie Lu, Ying Zhang, HanZheng Zhao, Qingshan Li, Ying Liu, YanZhen Zuo, Qian Xu, Hongyan Zuo, Yang Li, YuHong Li

**Affiliations:** 1 Department of Pathology, Chengde Medical College, Chengde, Hebei, China.; 2 Cancer Research Laboratory, Chengde Medical College, Chengde, Hebei, China.; 3 Department of General Surgery, The Second Affiliated Hospital of Harbin Medical University, Harbin, Heilongjiang, China.; 4 Department of oncology, The First Affiliated Hospital of Chengde Medical College, Chengde, Hebei, China.; 5 Beijing Institute of Radiation Medicine, Beijing, China.

**Keywords:** chronic stress, salbutamol, gastric cancer, β2-AR, ERK, epithelial-mesenchymal transition

## Abstract

Chronic stress induced by long-term anxiety and depression can promote the malignant progression of gastric cancer. β2-adrenergic receptor (β2-AR) is a critical mediator for chronic stress-induced multiple processes of tumor cells. However, the function of chronic stress in gastric cancer and its potential mechanisms *in vivo* and *in vitro*, especially at the cellular level, remain unknown. Here, we provide further evidence that chronic stress affected behavior and hypothalamus pituitary adrenal axis related hormone levels in mice. Furthermore, immunofluorescence showed that emotion affected the expression of epithelial-mesenchymal transition (EMT) markers in patients' tissues. To address this, salbutamol, a specific agonist of β2-AR, was utilized for simulating chronic stress and demonstrating the mechanism of stress in tumor progression at the molecular level both *in vivo* and *in vitro*. Salbutamol significantly induced EMT, migration and invasion via ERK (Extracellular-signal-regulated kinase) phosphorylation, and the effects were reversed by the β2-AR antagonist ICI-118,551. The promoting effects of salbutamol on EMT, migration and invasion were inhibited by phosphorylation inhibitor of ERK PD98059 *in vitro*. Analysis of xenograft models revealed that salbutamol significantly promoted tumor growth and adrenal volume, while ICI-118,551 inhibited these effects. In addition, salbutamol increased the expression of mesenchymal marker N-cadherin and decreased epithelial marker E-cadherin in transplanted tumor tissue. In conclusion, salbutamol simulates a chronic stress model, which promotes tumorigenesis of gastric cancer cells through β2-AR/ERK/EMT pathway.

## Introduction

Patients with malignant tumor will have various psychological stresses such as anxiety and depression 1. The body will be in a state of chronic stress after long-term exposure to negative emotional stimulation such as anxiety and depression. Chronic stress can change endocrine function by activating the sympathetic nervous system to release neurotransmitters such as norepinephrine and epinephrine 2. A recent study suggests that chronic stress acts as a risk factor and promotes the progression of multiple tumor types by facilitating epithelial-mesenchymal transition (EMT) and tumor cell growth 3.

The malignant progression of tumor is a sequential process, i.e. tumor cells proliferate continuously, migrate away from the primary focus and invade surrounding tissues and distant organs. In this process, the important role of EMT has been recognized 4. EMT is a highly conserved process, through which the cells lose epithelial characteristics and acquire mesenchymal characteristics, thus increasing the ability of tumor metastasis and recurrence, and leading to poor prognosis 5. As mentioned earlier, chronic stress can cause excessive release of stress-related hormones through the sympathetic nervous system and hypothalamus pituitary adrenal (HPA) axis. The two pathways have common target adrenergic receptors, which leads to poor prognosis and increased mortality in cancer patients 6; 7.

Study has shown that β2-adrenergic receptor (β2-AR) is a critical mediator for chronic stress-induced bio-behavioral changes of tumor cells 7. Our previous research found that β2-AR agonist isoproterenol promoted EMT of gastric cancer cells through STAT3-CD44 8. Other researchers have also found that isoproterenol, through the β2-AR-HIF-1α-Snail signaling pathway, affected the EMT of gastric cancer cells and then promoted the invasion and migration of gastric cancer 9. Another study investigated the effects of β2-AR activation on the anti-proliferation activities of trastuzumab. Their results revealed that activation of β2-AR triggered “targeting failure'' of trastuzumab in gastric cancer cells 10. The impact of chronic stress on tumor growth induced by β2-AR in humans has become increasingly concerning.

The research on the mechanism of gastric cancer needs to be carried out at the cellular level, and there is no chronic stress model in cells. Therefore, a method to simulate chronic stress* in vitro* is needed for β2-AR agonists. In this study, we first found that chronic unpredictable stress promoted depression-like behavior and affected the hormone levels of catecholamine. Then, we demonstrated the stress-activated effects of the β2-AR/ERK/EMT signaling pathway on the tumor growth of gastric cancer cells *in vitro* and *in vivo* by salbutamol, a specific agonist of β2-AR.

## Materials and methods

### Chronic unpredictable mild stress of mice

**Figure 1:**
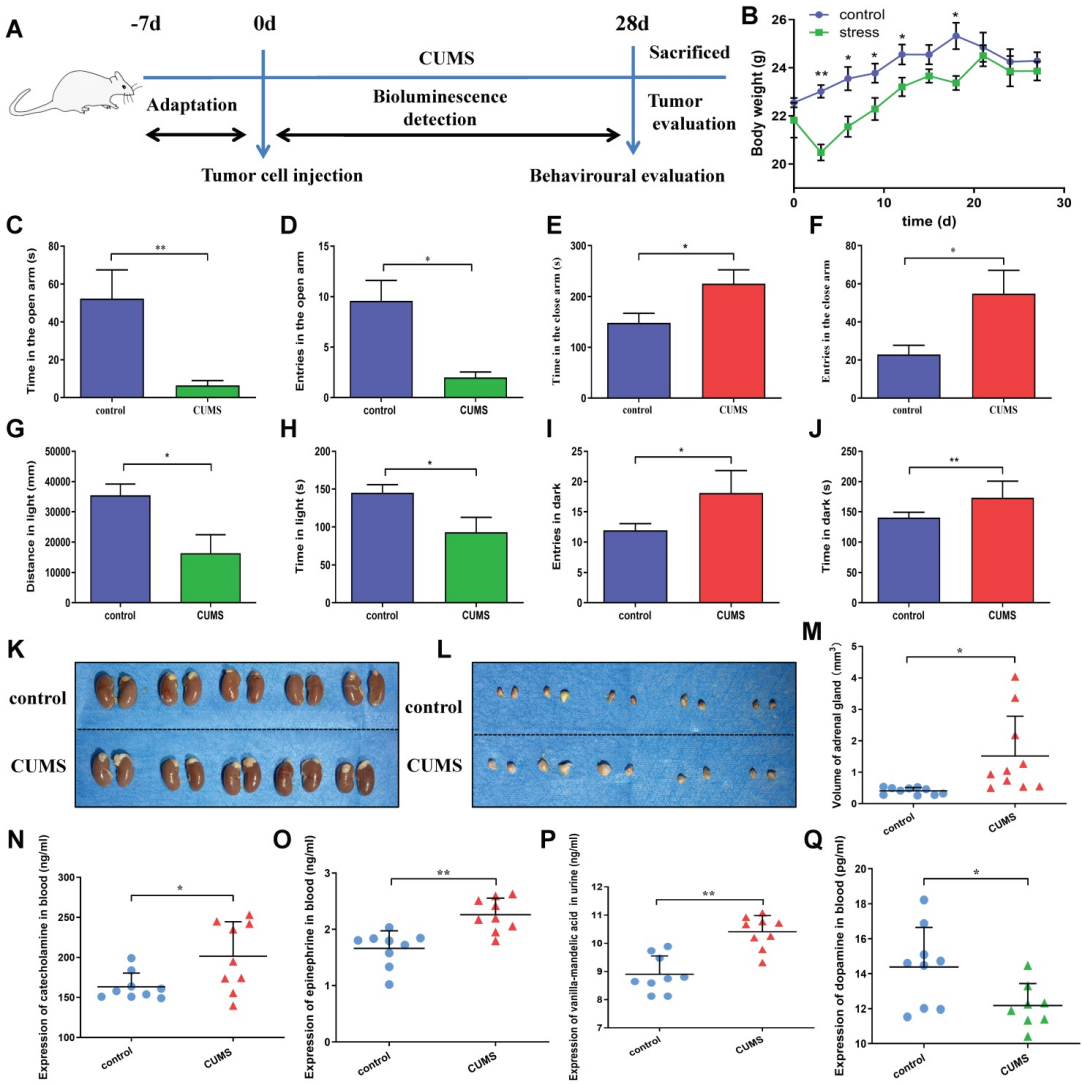
A: A schematic illustrates the timeline of experimental procedures, which include tumor cell inoculation, CUMS, behavioral evaluation and *in vivo* tumor detection. B: Effects of CUMS on body weight gain of mice is shown. C-F: Effects of chronic stress as evaluated by the elevated plus maze. Panels C and D show time in the open arms and entries in the open arms, respectively. Panels E and F show time and entries in the close arms, respectively. G-J: Effects of chronic stress as assessed by the light and dark box test. Panels G and H show total distance and time exploring the light area, respectively. Panels I and J show total entries in dark and time exploring the dark area, respectively. Data represent mean± SEM (n=6 for each group, *P<0.05, **P<0.01). K-L: Representative adrenal gland images are displayed. M: The adrenal gland diameters of two groups were measured. N: Mouse serum catecholamine levels. O: Mouse serum epinephrine levels. P: Mouse urine VMA levels. Q: Mouse serum dopamine levels. Data represent mean± SD (*P<0.05, **P<0.01).

The experimental procedure is shown in Fig. 1A. After a week of acclimation, the mice were inoculated subcutaneously to establish tumor-bearing models. The Chronic unpredictability mild stress (CUMS) was designed according to the methods described previously with a slight modification 11. The CUMS protocol consisted of various stressors for 4 weeks: (1) food deprivation for 24 h, (2) water deprivation for 24 h, (3) swimming in water at 42℃ for 5 min, (4) swimming in water at 4℃ for 5 min, (5) damp sawdust for 24 h, (6) overnight illumination, (7) tails clamped for 1 min, (8) restraint for 5 min, (9) making noise for 5 min, and (10) cage tilting (45◦) for 24 h. Stressors were managed randomly at any time every 1-2 days. The stress sequences changed weekly to make the stress program unpredictable. These stressors were randomly assigned for 4 weeks. After the behavioral test, the mice were sacrificed. Blood and urine samples were obtained.

### Elevated plus maze

The mice were placed in the center of a cross maze, which was made of two open arms and two closed arms and 60 cm above the ground. All mice were placed into the center of the maze facing the same open arm and then were allowed to freely explore the maze for a testing period of 5 min. Meanwhile a digital camera recorded their activities. The observers were blinded to the treatment of the mice. The number of entries and time spent in open and closed arms were recorded for analysis.

### Light and dark box test

Light-dark box consisted of two compartments. One compartment was fully opaque, while the other was lit from the compartment ceiling by a 20 W bulb. A small opening in the partition wall allowed free passage between the light and dark compartments. Mice were individually placed in the dark side of the light-dark box and were allowed to move freely for 5 min. The observers were blinded to the treatment of the mice. A video camera recorded the behavior of each mouse for later analysis of behavior crossing between the dark and the light compartment.

### ELISA assay

After 28 days, we collected the serum of mice using procoagulant blood collection and the urine by mouse metabolic cage. Mice blood and urine samples were subjected to 15 min of centrifugation at 3000rpm under 4 °C. 300-500 μl serum was centrifuged from the blood of each mouse. Mouse serum catecholamine, epinephrine and dopamine, as well as mouse urine vanillylmandelic acid (VMA) levels were examined using the corresponding ELISA kits (Mlbio biotech company, Shanghai, China) in accordance with specific protocols. The absorbance at 450 nm was measured on the microplate reader.

### Human tissue specimens

Gastric cancer patients were from the First Affiliated Hospital of Chengde Medical University. According to the Zung Self-Rating Depression Scale index scores, the patients were divided into those with low levels of depression (SDS Index: 53-62) and those with high levels of depression (SDS Index ≥63) 12.

### Immunofluorescent staining

Gastric tissues sections were fixed in 4% paraformaldehyde for 30 min and then permeabilized in 0.2% Triton X-100 in PBS for 10 min. The sections were co-stained with anti-E-cadherin (1:200) and anti-N-cadherin (1:200) antibody. Then, Alexa Fluor ® 488-conjugated AffiniPure goat anti-mouse IgG (abs20013; Absin Biotechnology, Inc, Shanghai, China) and Alexa Fluor® 594-conjugated AffiniPure goat anti-rabbit IgG (ab150080; Abcam, Cambridge, MA, UK) were used at 1:500 dilution. The nuclei were stained using DAPI (Vector Laboratories Inc, Burlingame, CA). Fluorescence images were collected with a laser scanning confocal microscope (Leica, Solms, Germany).

### Cell culture

Human gastric cancer cell lines MGC803 and SGC-7901 were provided by Chinese Medical Sciences University. MGC803-Luciferase-Puro was provided by Umibio Co. Ltd (Shanghai, China). These cells were cultivated with the Roswell park memorial institute 1640 (RPMI-1640) medium or Dulbecco's modified eagle medium (DMEM, Gibco Company, St. Louis, MO, USA) containing 10% fetal bovine serum (FBS, Gibco Company, St. Louis, MO, USA) in an incubator with 5% CO_2_ at 37˚C.

### Quantitative real time-Polymerase Chain Reaction (qRT-PCR)

Trizol was utilized for extracting total RNA. Then, 1 µg total RNA was reverse transcribed into cDNA using ABScript Ⅱ RT Master Mix for qPCR kit (ABclone, China). All PCR primers sets are listed in Table S1. The 2×Universal SYBR Green Fast qPCR Mix Kit (ABclone, China) was utilized for qRT-PCR. GAPDH was the internal reference. The 2^-ΔΔCt^ method was adopted to determine relative mRNA expression.

### Western blot analysis

The cold radio immunoprecipitation assay (RIPA) buffer (Thermo Scientific, Rockford, IL, USA) was utilized for cell lysis. The bicinchoninic acid (BCA) Protein Assay kit (Beijing Solarbio Science & Technology Co., Ltd., China) was used for determining protein concentration. After dodecyl sulfate, sodium salt-polyacrylamide gel electrophoresis (SDS-PAGE), proteins were transferred to the polyvinylidene fluoride (PVDF) membranes. Then, the membrane was subjected to overnight incubation using primary antibodies at 4°C, and the information of the primary antibodies is available in Table S2. Afterwards, horseradish peroxidase (HRP)-labeled secondary antibodies (Abcam, Cambridge, MA, UK) were further used to incubate the membranes for 2 h. In addition, the enhanced chemiluminescence substrate (Pierce, Rockford, IL, USA) was used for color development of protein blots. Image analysis software was employed for visualization.

### Transwell assay

The gastric cancer cells were collected and resuspended in serum-free RPMI-1640 medium at a concentration of 4×10^5^ cells/ml. Then, the cell suspension was seeded into the upper chambers (200 μl/well), and the bottom chambers were filled with RPMI-1640 containing 10% FBS (1 ml/well). We used CORNING (Tewksbury, MA, UK) Transwell chambers (Lot 3422) to quantify cell migration. For the Transwell invasion assay, CORNING transwell chambers (Lot 354480) were used, in which matrigel was pre embeded in the upper chambers. After cultured at 37°C for 24 h, the cells that had not penetrated the polycarbonate membrane were wiped off with a cotton swab. The membrane was removed, fixed in 4% paraformaldehyde and stained with crystal violet solution. The number of migrated and invaded cells was determined by counting cells in 5 fields of view (Olympus Corp, Tokyo, Japan).

### *In vivo* tumorigenicity assay

A total of 36 male BALB/c nude mice (age 6 weeks, body weight 16-20g) were provided by Huafukang Bioscience Co., LTD (Beijing, China) and housed within the individually ventilated caging systems. The mice were given subcutaneous injection of MGC803-luc gastric cancer cell suspension (200 μl; 1×10^6^ cells/ml) in the posterior region. Tumor progression *in vivo* was tracked with bioluminescence once a week using an IVIS Lumina II (Perkin Elmer) imaging system by measuring luciferase activity after an intraperitoneal injection of 10ul/g D-luciferin (15 mg/ml). Then mice were sacrificed for tumor evaluation after 28 days. Primary tumors were measured by caliper, and volume was calculated by the formula of (length * width^2^)/2.

### Statistical analysis

All experiments were carried out in triplicates at least. The results of behavioral experiments were expressed as the mean ± SEM. Results of cell experiments were expressed as mean ± SD. Student's t-test and One-way ANOVA were utilized for statistically analyzing data. A difference of P<0.05 indicated statistical significance, as marked with asterisks in all figures.

## Results

### Chronic unpredictable stress affects the behavior and hormone levels in mice

The schematic timeline showed the experimental procedures (Figure 1A). After a week of acclimation, the mice were subcutaneously inoculated with human gastric cancer cells. CUMS was used to construct the chronic stress model. After 4 weeks, the mice were tested for behavioral and hormonal changes. Meanwhile, tumor growth was also assessed.

To investigate the effects of CUMS on mice, we detected the changes in the body weight gain of mice. We observed that nude mice consumed less food, drank less water, showed reduced activity and lost weight in the persistent CUMS. As shown in Figure 1B, CUMS induced weight loss in mice after 3 days. However, the change was not obvious after 18 days. There are certain reasons why the body weight of the stressed mice caught up with the mice in the control group during the later stage. First reason is stress itself. Stress as we know, is a challenge to the body's natural homeostasis. The body usually responds to stress by generating physiological responses to regain the balance lost due to the influence of the stressors 13. As previous studies have shown, severe stressors can induce hyperphagia; which is referred to as stress-induced hyperphagia 14. Secondly, stress-related activation of the HPA axis can alter glucose metabolism, promote insulin resistance and influence multiple appetite-related hormones and hypothalamic neuropeptides 15. In addition, the HPA axis stimulates the release of adrenocorticotropic hormone (ACTH), and subsequently triggers the production of glucocorticoids (GCs), which is related to obesity and metabolic disease 16. These findings were also confirmed in a study with 339 adults, so it has been determined that chronic stress can further lead to weight gain over time 17.

To further investigate the effects of CUMS-induced anxiety-like behaviors of mice, we performed the elevated plus maze test and light and dark box test, which are widely used tests for measuring CUMS-induced anxiety-like behaviors in mice 18.

Figure 1C-D depicts total time spent and numbers of entries in open arms. There was a significant inhibitory effect of CUMS on the activity of mice in open arms. Figure 1E-F represents time spent and numbers in close arms. Similar to open arms, there was a significant effect of CUMS on the activity of mice in close arms. Figure 1G-H shows that the distance and time spent in the light compartment were reduced in CUMS. Similar to the light compartment, it significantly increased the time and entries of mice in the dark box (Figure 1I-J). Collectively, these findings showed a significant effect of CUMS on anxiety-like behaviors.

Interestingly, we also found that the adrenal glands of CUMS group were larger than that in the control group in mice. It suggests that the HPA axis remains active in CUMS state (Figure 1K-M). So, we further detected the adrenal glands related hormone levels.

The concentrations of serum catecholamine and epinephrine in CUMS group were demonstrably higher than those in the control group. Vanillylmandelic acid (VMA) is the final metabolite of catecholamine such as epinephrine and norepinephrine. We found that urine VMA level in CUMS group was also significantly higher than that in the control group. In this study, we further tested the change of serum dopamine levels. The dopamine levels in CUMS group were significantly decreased compared with those in the control group. (Figure 1N-Q). Overall, these results indicate that CUMS has a significant effect on HPA axis related hormone levels.

### Depression affects the expression of EMT markers in patients' tissues

EMT plays a crucial role in cancer progression, which enhances cancer cell invasion and aggressive ability 19. In EMT, there is always downregulated epithelial markers (E-cadherin) and upregulated mesenchymal markers (N-cadherin) 20. Previous study has shown a close relationship between EMT and cancer stemness 21. However, the effect of CUMS on EMT of tumor cells is unclear.

**Figure 2:**
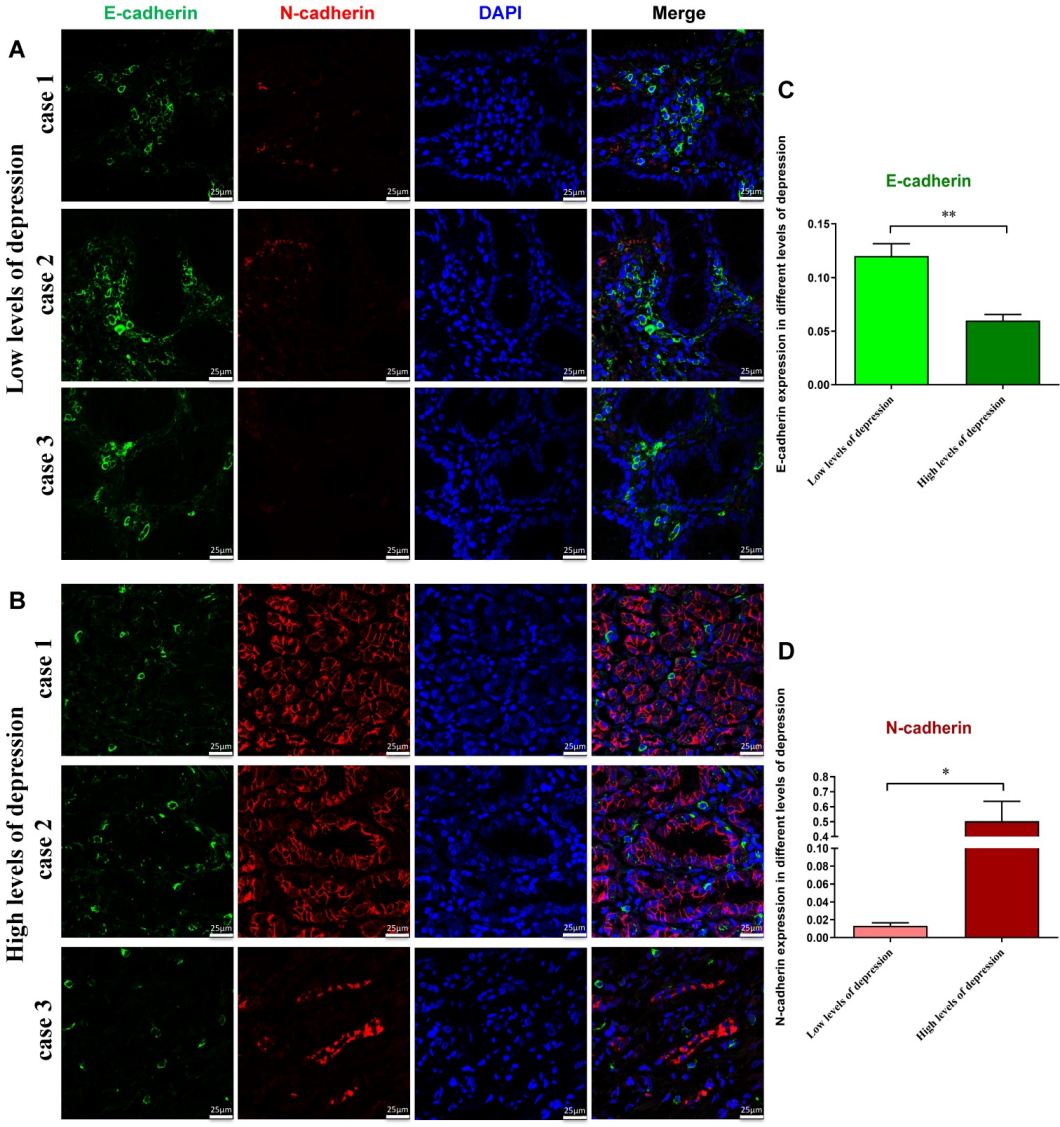
Immunodetection of E-cadherin and N-cadherin proteins in gastric cancer patients with the low (A) or high levels of depression (B). Fluorescent imaging was obtained with a confocal laser scanning microscope (Bar=25μm). We counted the numbers of E-cadherin^+^ (C) and N-cadherin^+^ (D) in different levels of depression respectively. C-D: E-cadherin fluorescence signal intensity in the group with low levels of depression was higher than in those with high levels of depression, whereas N-cadherin was lower in the group with low levels of depression.

According to the SDS scores, the patients were divided into those with low levels of depression and those with high levels of depression. To further determine possible alterations of EMT process of tumor cells in patients, fluorescent immunostaining was performed. The results showed that the E-cadherin fluorescence signal intensity in the group with low levels of depression was higher than that in the group with high levels of depression, whereas N-cadherin was substantially decreased in the group with low levels of depression (Figure 2A-D). These results further suggest that high level depression has promotive effects on EMT of tumor cells.

### Chronic stress promotes migration and invasion of gastric cancer cells via β2-AR/ERK/EMT pathway* in vitro*

As mentioned above, CUMS activates the HPA axis and affects the expression of related hormones. β2-AR is a critical mediator for chronic stress-induced development and progression of gastric cancer cells. However, to explore the mechanism *in vitro*, it is necessary to establish a chronic stress model in cells. Therefore, salbutamol, a specific agonist of β2-AR, was selected in this study to simulate chronic stress in cells.

**Figure 3:**
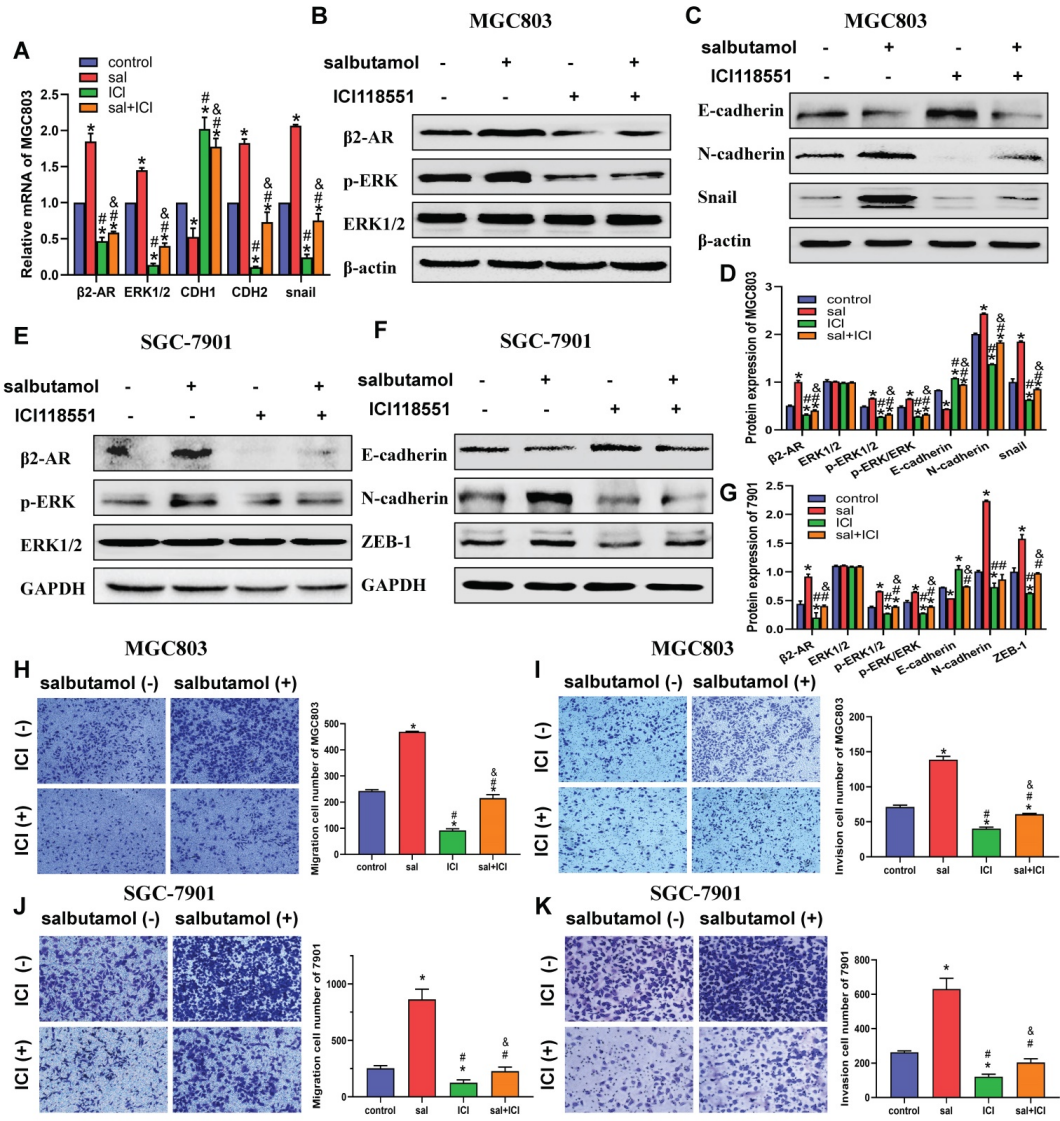
MGC803 and SGC-7901 cells were treated with 16 μM salbutamol and/or 10 μM ICI-118,551. A: The mRNA expression of *β2-AR*, *ERK1/2*, *CDH1*, *CDH2* and *Snail* were detected by qRT-PCR. B: The expression of β2-AR, phosphorylation ERK, E-cadherin, snail and N-cadherin were analyzed by Western blotting. C: The protein expression of EMT markers in MGC803. D: The relative protein expression in MGC803 cells were analyzed. E: The protein expression of β2-AR, phosphorylation ERK and ERK in SGC-7901. F: The protein expression of EMT markers in SGC-7901. G: The relative protein expression in SGC-7901 cells were analyzed. Representative images of migration (H) and invasion (I) in MGC803 are shown on the left and the quantification results of five randomly selected fields are shown on the right. Representative images of migration (J) and invasion (K) in SGC-7901 are shown. (*: *P*<0.05, compared with the control group; #: *P*<0.05, compared with the salbutamol group; &: *P*<0.05, compared with the ICI-118,551 group)

In the present study, we first performed qRT-PCR analysis to detect *β2-AR* and *ERK* mRNA expression in gastric cancer cell lines after salbutamol treatment. Salbutamol stimulation increased *β2-AR* and *ERK* mRNA levels. However, ICI-118,551, the selective antagonist of β2-AR, remarkably decreased the mRNA expression of *β2-AR* and *ERK*. In addition, qRT-PCR results showed that salbutamol upregulated mesenchymal markers, *CDH2* (*N-cadherin*) and* Snail* expression, and decreased the expression of epithelial marker *CDH1* (*E-cadherin*) at transcription levels (Figure 3A).

To further address the role of salbutamol on the β2-AR/ERK pathway and EMT, Western-blot analysis was performed to examine the changes in protein expression. Our results indicated that salbutamol stimulation resulted in a significant upregulation of β2-AR and ERK phosphorylation, whereas ICI-118,551 treatment reversed this increase. (Figure 3B, 3D) Additionally, Western-blot analysis showed that at protein levels, salbutamol upregulated the expression of N-cadherin and Snail, and downregulated the expression of E-cadherin (Figure 3C, 3D). It showed a clear role of the salbutamol-mediate EMT process. Similar results were observed in SGC-7901 cells at protein levels. (Figure 3E-G). These observations suggest that salbutamol promotes EMT of gastric cancer cells via β2-AR/ERK.

Furthermore, transwell assay was performed to detect salbutamol and ICI-118,551 effects on the migration and invasion of gastric cancer cell. Our results showed that salbutamol increased the migration and invasion while ICI-118,551 impaired the migration and invasion of gastric cells. (Figure 3H-K) Collectively, these findings suggest that chronic stress promotes migration and invasion of gastric cancer cells via β2-AR/EMT pathway.

**Figure 4:**
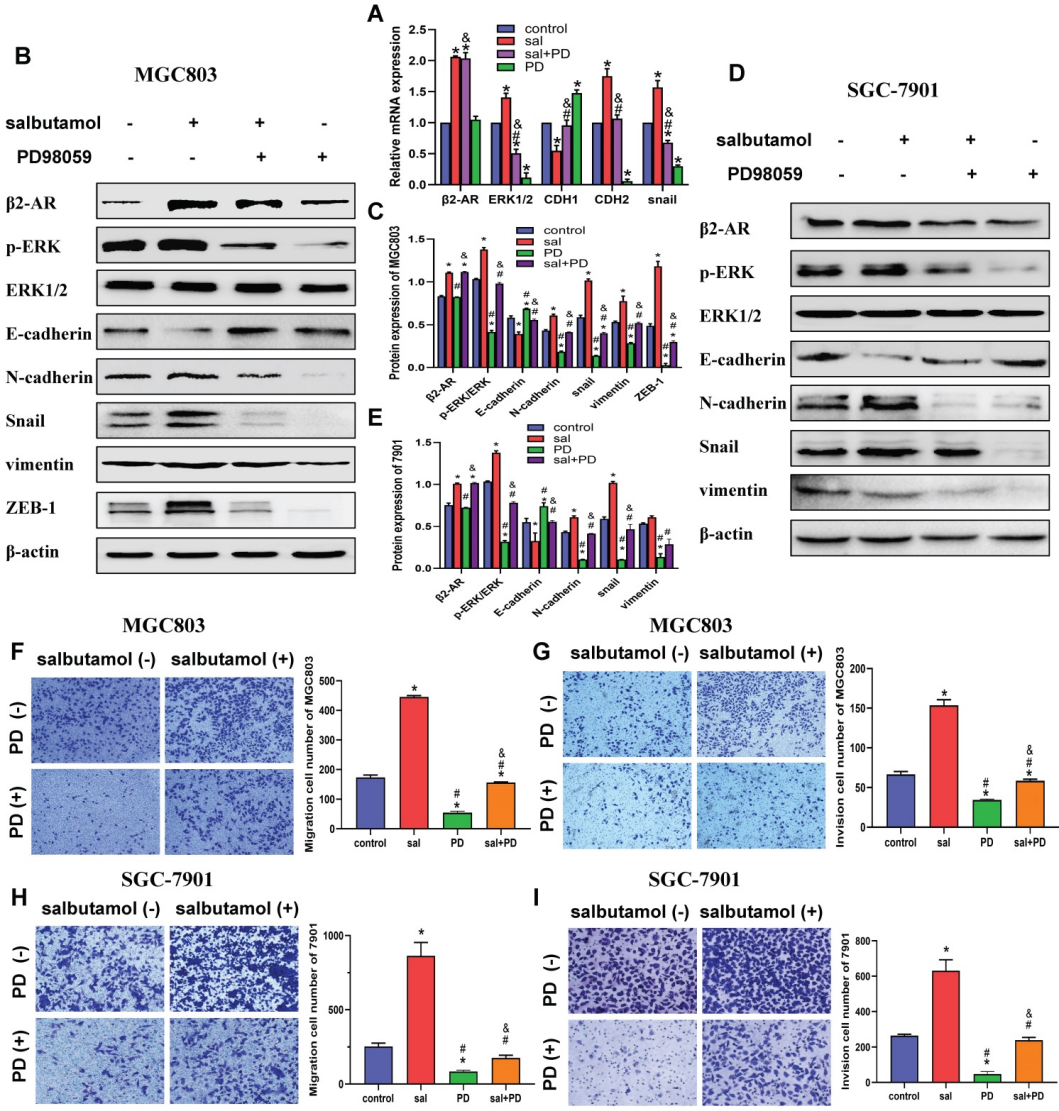
MGC803 and SGC-7901 cells were pretreated with 50 μM PD98059 for 2 h and then incubated in 0 or 16 μM salbutamol for an additional 12 h. A: The mRNA expression in MGC803. B: The expression of β2-AR, phosphorylation ERK and EMT markers in MGC803. C: The relative protein expression in MGC803. D: The expression of β2-AR, phosphorylation ERK and EMT markers in SGC-7901. E: The relative protein expression in SGC-7901. Representative images of migration (F) and invasion (G) in MGC803 are shown on the left and the quantification results of five randomly selected fields are shown on the right. Representative images of migration (H) and invasion (I) in SGC-7901 are shown. (*: *P*<0.05, compared with the control group; #: *P*<0.05, compared with the salbutamol group; &:*P*<0.05, compared with the ICI-118,551 group)

We then investigated whether ERK is a potential regulator involved in salbutamol-induced EMT in gastric cancer cells. MGC803 and SGC-7901 cells were pretreated with PD98059, a phosphorylation inhibitor of ERK, before treating with salbutamol for 12 h. qRT-PCR analysis showed that PD98059 pretreatment reversed the activation of β2-AR and process of EMT by salbutamol. (Figure 4A) Western-blot analysis indicated that salbutamol increased β2-AR and p-ERK activation, whereas PD98059 pretreatment reversed the increases. Whether the inhibition of phosphorylation ERK can affect the process of EMT and their association with β2-AR activation were further explored. Our results showed that PD98059 decreased the expression of EMT-related proteins, showing upregulation of epithelial marker (E-cadherin) and downregulation of mesenchymal markers (N-cadherin, Snail, vimentin, ZEB-1). However, salbutamol stimulation significantly reversed the decrease of EMT induced by PD98059 (Figure 4B-E). Our observations indicate that there is a clear role for the ERK pathway in salbutamol-mediate EMT induction.

Additionally, transwell analysis was performed to detect the effects of ERK phosphorylation inhibitor on migration (Figure 4F, 4H) and invasion (Figure 4G, 4I) of gastric cancer cells. Our results showed that PD98059 inhibited migration and invasion of gastric cancer cells. However, this inhibitory effect could be partially reversed by β2-AR agonist salbutamol. Collectively, these findings suggest that chronic stress promotes migration and invasion of gastric cancer cells via β2-AR/ERK/EMT pathway.

### *In vivo* analysis of salbutamol and ICI-118,551 in regulating tumor growth of gastric cancer cells

**Figure 5:**
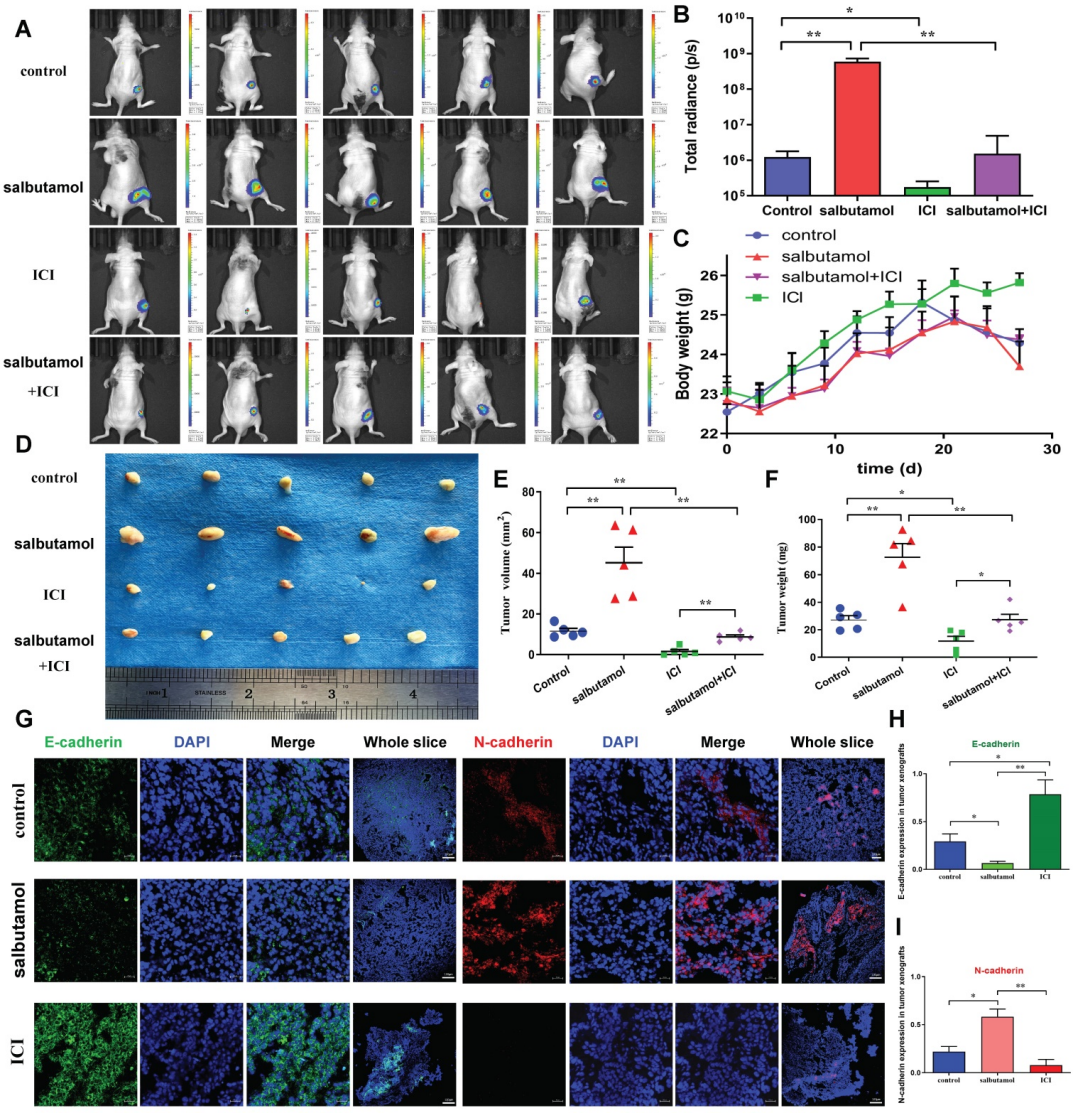
Mice were treated with salbutamol (5 mg/kg/d) and/or ICI-118,551 (0.2 mg/kg/d). A: After 2 weeks, representative images of bioluminescence from mice with MGC803-Luc tumors in 4 groups. B: Quantification of bioluminescence from mice with MGC803-Luc tumors. C: The weight curve of nude mice after treatment with salbutamol or ICI-118,551 is shown. D: Representative picture of BALB/c nude mice receiving subcutaneous inoculation of MGC803 cells. E: Volume of tumor xenografts resected from BALB/c nude mice. Data represent mean ± SD (n=5 for each group,*P<0.05, **P<0.01). F: Weight of tumor xenografts resected from BALB/c nude mice. Data represent mean ± SD (n=5 for each group, *P<0.05, **P<0.01). G: Immunodetection of E-cadherin and N-cadherin proteins in nude mice treated with salbutamol or ICI-118,551. Representative pictures (Bar=20 μm) and the whole slices (Bar=100 μm) of fluorescence are shown. We counted the numbers of E-cadherin^+^ (H) and N-cadherin^+^ (I) in the tumor xenografts of mice respectively. H-I: The expression of tissue mesenchymal marker N-cadherin was up-regulated, and the expression of epithelial marker E-cadherin was down-regulated in the salbutamol treated group. N-cadherin was down-regulated, and E-cadherin was up-regulated in the ICI-118,551 treated group. Data represent mean ± SD (n=3 for each group, *P<0.05, **P<0.01).

We first validated whether salbutamol affects tumor growth *in vivo*. *In vivo* imaging analysis showed that salbutamol increased the ability of tumor growth. Moreover, ICI-118,551 inhibited the tumor growth in nude mice (Figure 5A-B). During the observation of changes in mice, nude mice consumed less food, drank less water, showed reduced activity and lost weight in the salbutamol treatment group (Figure 5C). Next, we evaluated the effects of salbutamol and ICI-118,551 on tumor volume and weight. The tumor volume and weight of the salbutamol group was remarkably higher than that of the control group. By contrast, the tumor volume and weight of the ICI-118,551 group was smaller than that of the control group (Figure 5D-F). Immunofluorescent staining of tumor specimens revealed that salbutamol increased the EMT of tumor cells, and ICI-118,551 inhibited the EMT of gastric cancer cells* in vivo* (Figure 5G-I). Collectively, these findings suggest that chronic stress promotes tumorigenesis of gastric cancer cells via the EMT pathway.

## Discussion

Recently, the role of chronic stress in the development of cancer is gradually being studied and widely recognized 22. Gastric cancer is the most common malignancy worldwide 23. Its incidence and mortality rank the fifth and the third of global cancer, respectively 24. The nervous system has been recently shown to exert an impact on gastric cancer by many nervous system-associated factors, which participate in the regulation of many aspects of gastric cancer such as cell proliferation, metastasis and recurrence.

The study on the underlying mechanism of chronic stress has revealed that the HPA axis is activated, which further leads to the release of catecholamines from the adrenal gland, sympathetic nerve terminals and the brain, including norepinephrine, epinephrine and dopamine 25. Using monkey models, Levine and Wiener et al. demonstrated the physiological and behavioral responses of monkeys after long-term maternal and child separation and the increase of plasma HPA axis related hormone levels 25. HPA axis related hormones are involved in gastric cancer progression mainly through binding to β2-AR and further activating several downstream signaling pathways 27.

Research on the mechanism of gastric cancer needs to be carried out at the cellular level. Thus, chronic stress in cells simulated by catecholamines or a kind of β2-AR agonist *in vitro* is needed. Isoproterenol has been used in previous experiments, but it has a strong stimulative effect on both β1 and β2 receptors 28. Salbutamol is a specific β2-AR agonist, and its chemical structure is similar to that of isoproterenol. Compared with isoproterenol, salbutamol is more β2 selective. More importantly, increasing heart rate effect of salbutamol is lower than that of isoproterenol, and oral administration of salbutamol is effective with a longer duration 29.

Salbutamol is not an HPA axis related hormone secreted by the body itself. Theoretically speaking, it can activate β2-AR only, and it should not affect the positive and negative feedback of the related hormones. Based on the findings of this study, we speculate that salbutamol has the potential to be an effective mimic drug of chronic stress. The β2-AR blockers, as a routine clinical drug for cardiovascular diseases, have a broad and clear effect. However, the direct use of β2-AR blockers in clinical psychotherapy of tumor patients will be limited due to their adverse reactions. Therefore, there is an urgent need to explore new targets that can block the signaling pathway of chronic stress.

ERK belongs to the MAPK (mitogen activated protein kinase) family. It plays a role in the signal cascade and the transmission of extracellular to intracellular signals. ERK can be activated by cytokines, hormones and neurotransmitters. After phosphorylation, ERK is transferred to the nucleus and regulates various transcription factors, thus regulating cell metabolism and function. It is reported that the ERK/ MAPK signaling pathway plays an important role in tumor proliferation, invasion and metastasis 30. Liu et al. found that death domain associated protein-6 promoted the proliferation and migration of ovarian cancer cells by activating the ERK signaling pathway 30. Using a mouse model of metastatic xenotransplantation of human ovarian cancer cells, Sung et al. found that knockout of γ-GABA receptor reduced cell migration and invasion by reducing ERK activation 31. This study is a powerful supplement to the theory of chronic stress-induced migration and invasion of gastric cancer cells by ERK/EMT signaling pathway.

Our study has some limitations: 1. Although salbutamol acts as a selective β2-AR agonists, it mimics the effects of chronic stress state theoretically. However, it needs to be further detected whether the effects of salbutamol are consistent with the changes in cells under chronic stress. 2. The chronic stress state of animals includes complex psychological changes, which can lead to changes of hormone levels and behaviors. So, whether a selective β2-AR agonist can fully model this pathological change also needs to be further explored.

**Figure 6:**
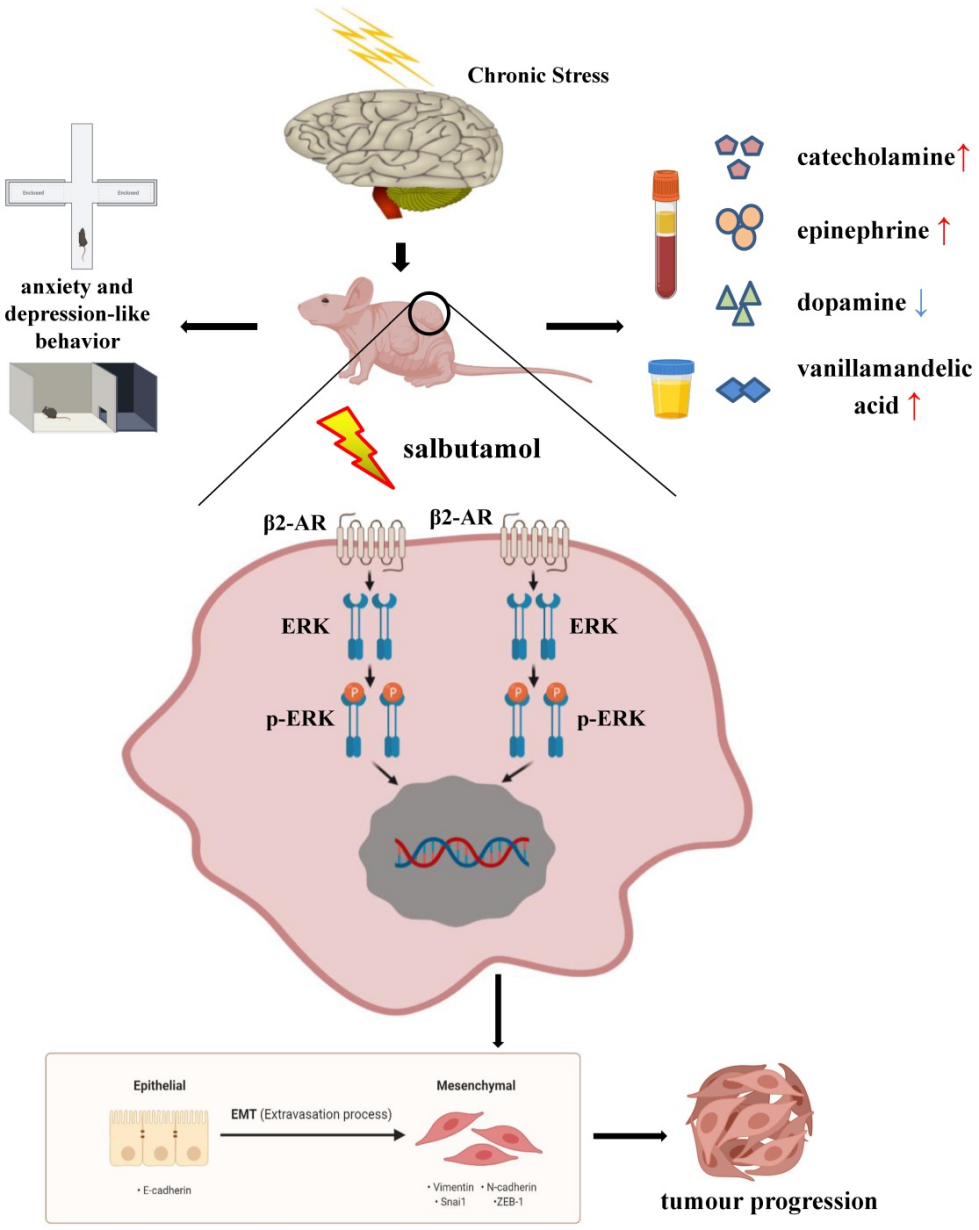
Schematic representation summarizes the results from the present study. Chronic stress changes the behaviors of anxiety and depression in mice. At the same time, chronic stress causes the changes of HPA axis related hormones in blood and urine. Chronic stress can increase the serum concentrations of catecholamine, epinephrine and urine VMA, a final metabolite of catecholamine, and decrease the serum concentration of dopamine. Salbutamol, a specific agonist of β2-AR, was selected in this study to simulate chronic stress in cells. Salbutamol can recognize and activate β2-AR, which subsequently activate its down-stream target ERK and further promote ERK phosphorylation. Phosphorylation of ERK functioned as a positive regulator of EMT under chronic stress, which subsequently promoted cancer progression of human gastric cancer cells.

Taken together, chronic stress affected the behavior and HPA axis related hormone levels in mice. Salbutamol, a selective β2-AR agonist, recognized and activated β2-AR, which subsequently activated its down-stream target ERK. ERK activation functioned as a positive regulator of EMT under chronic stress, which subsequently promoted cancer progression of human gastric cancer cells. (Figure 6) Our findings provide evidence and basis for a better understanding of the mechanism connecting psycho-social and biobehavioural influences on gastric cancer pathogenesis and suggest that pharmacological interventions targeting β2-AR/ERK/EMT signaling can potentially be used to ameliorate the effects of chronic stress on gastric cancer progression.

## Supplementary Material

Supplementary tables.
