# Supplementary material for: Chronic stress model simulated by salbutamol promotes tumorigenesis of gastric cancer cells through β2-AR/ERK/EMT pathway
Source: J Cancer. 2022 Jan 1;13(2):401–12. doi: 10.7150/jca.65403 (PMC8771504; doi:10.7150/jca.65403)
Supplement: Supplementary file 1 — Supplementary tables. [file jcav13p0401s1.pdf]

Table S1. Primer sequences used in this study

Gene	Sequences
ERK1/2	5’-ATGGTGTGCTCTGCTTATGATA-3’ (Sense)<br>5’-TCTTTCATTTGCTCGATGGTTG-3’ (Antisense)
β2-AR	5’-CCATTGATGTGCTGTGCGTC-3’ (Sense)<br>5’-CCGGGCCTTATTCTTGGTCA-3’ (Antisense)
CDH1	5’-GATAGAGAACGCATTGCCACATA-3’ (Sense)<br>5’-ATTTTCCATGACAGACCCCTTAA-3’ (Antisense)
CDH2	5’-TGTTTGTCCTTACTGTTGCTGC-3’ (Sense)<br>5’-TTCTTCTTGGCGAATGATCTTAG-3’ (Antisense)
snail	5’-GCTCTGCCACCCTGGGACTC-3’ (Sense)<br>5’-CCTCGCTGCCAATGCTCATCTG-3’ (Antisense)
GAPDH	5’-GAAGGTCGGAGTCAACGGAT-3’(Sense)<br>5’-CTGGAAGATGGTGATGGGATT-3’ (Antisense)

Table S2. List of primary antibodies used.

Antibody	Source	Catalog Number Host	Host	Dilution
β2-AR	abcam	ab182136	mouse	1:1000
ERK1/2	Huaan	#ET1601-29	rabbit	1:1000
p-ERK1/2	Huaan	#ET1603-22	rabbit	1:1000
E-cadherin	Huaan	#ET1607-75	rabbit	1:1000
N-cadherin	Huaan	#ET1607-37	rabbit	1:1000
snail	Huaan	#ER1706-22	rabbit	1:1000
vimentin	Huaan	#M0401	mouse	1:1000
ZEB-1	Cell Signaling Technology	#3396	rabbit	1:1000
β-actin	Cell Signaling Technology	#58169	mouse	1:10000
GAPDH	Abcam	ab8245	mouse	1:10000
